# The patient-specific mouse model with Foxg1 frameshift mutation uncovers the pathophysiology of FOXG1 syndrome

**DOI:** 10.21203/rs.3.rs-2953760/v1

**Published:** 2023-06-02

**Authors:** Jaein Park, Ji Hwan Moon, Holly O’Shea, Dongjun Shin, Seon Ung Hwang, Liwen Li, Hyojong Lee, Elise Brimble, Jae Lee, Stewart Clark, Soo-Kyung Lee, Shin Jeon

**Affiliations:** University at Buffalo; University at Buffalo; University at Buffalo; University at Buffalo; University at Buffalo; University at Buffalo; Sungkyunkwan University; Invitae; University at Buffalo; University at Buffalo; University at Buffalo; University of Pennsylvania

## Abstract

Single allelic mutations in the gene encoding the forebrain-specific transcription factor FOXG1 lead to FOXG1 syndrome (FS). Patient-specific animal models are needed to understand the etiology of FS, as FS patients show a wide spectrum of symptoms correlated with location and mutation type in the FOXG1 gene. Here we report the first patient-specific FS mouse model, Q84Pfs heterozygous (Q84Pfs-Het) mice, mimicking one of the most predominant single nucleotide variants in FS. Intriguingly, we found that Q84Pfs-Het mice faithfully recapitulate human FS phenotypes at the cellular, brain structural, and behavioral levels. Importantly, Q84Pfs-Het mice exhibited myelination deficits like FS patients. Further, our transcriptome analysis of Q84Pfs-Het cortex revealed a new role for FOXG1 in synapse and oligodendrocyte development. The dysregulated genes in Q84Pfs-Het brains also predicted motor dysfunction and autism-like phenotypes. Correspondingly, Q84Pfs-Het mice showed movement deficits, repetitive behaviors, increased anxiety, and prolonged behavior arrest. Together, our study revealed the crucial postnatal role of FOXG1 in neuronal maturation and myelination and elucidated the essential pathophysiology mechanisms of FS.

## Introduction

Foxg1 is the forkhead family transcription factor whose expression is induced as the telencephalon begins to form in embryos ^1^. The elimination of the Foxg1 gene results in a striking loss of the forebrain volume ^2^, highlighting the crucial role of Foxg1 in forebrain generation.

The cortex comprises six cortical layers occupied by distinct subtypes of cortical projection neurons produced from the neural progenitors within the dorsal telencephalon ^3^. In addition to excitatory projection neurons, the cortex contains inhibitory GABAergic interneurons that are generated in the ventral telencephalon and migrate tangentially to the developing cortex ^4^. Foxg1 plays a critical role in multiple steps of cortex development. Foxg1 is important for properly patterning the telencephalon at early stages ^5–7^. In neural progenitor cells, Foxg1 is required for the proliferation and maintenance of the progenitor population by blocking cell cycle exits and premature neuronal differentiation ^2,5,8,9^. In cortical neurons, Foxg1 plays a crucial role in differentiation, migration, subtype specification, and axon projection ^10–14^. The specific and complete elimination of Foxg1 in postmitotic cortical projection neurons results in inverted cortical layers ^11^, indicating that Foxg1 is needed for proper cortical lamination. Notably, deleting only a copy of the Foxg1 gene in cortical projection neurons is sufficient to attenuate the production of upper-layer neurons, changing the composition of cortical layers ^11^. Deleting an allele of the Foxg1 gene leads to reduced cortical interneurons ^15^, while the complete inactivation of Foxg1 in cortical interneurons interferes with their proper integration into the cortical layer ^16^. Therefore, Foxg1 coordinates the orderly production, migration, and integration of glutamatergic excitatory neurons and GABAergic inhibitory interneurons in the developing cortex. Importantly, these studies highlight that both copies of the Foxg1 gene are required for building the functional cortex.

In mice, neuronal migration and layer formation are largely completed by birth, and glial cells begin to emerge ^3^. Oligodendrocyte precursor cells (OPCs) in the cortex proliferate and differentiate into myelinating oligodendrocytes (OLs) after birth, and the myelination continues to adulthood ^17^.

In humans, the pathogenic variants in the FOXG1 gene lead to the debilitating neurodevelopmental disorder collectively termed FOXG1 syndrome (FS) ^18,19,19–23^. Most FS is caused by de novo mutations in a single allele of the FOXG1 gene. FS patients exhibit structural brain abnormalities, such as microcephaly and corpus callosum agenesis, and a delay in oligodendrocyte differentiation and myelination. FS is also characterized by severe intellectual disability, hyperkinetic-dyskinetic movement disorder, irritability, and epilepsy. Many FS patients have autistic features, such as repetitive movements, poor social interaction skills, and a near absence of verbal speech and language development. Hence, FS belongs to the autism spectrum disorder (ASD). Currently, the molecular and cellular mechanisms leading to the pathology of FS remain elusive.

The crucial involvement of FOXG1 in forebrain development and human pathogenesis raises several questions. First, what are the cellular changes triggering FS symptoms? Second, what are the molecular changes leading to FS pathology? Third, what is the role of FOXG1 in myelination, and how does the disturbed myelination in FS contribute to FS clinical manifestation?

Notably, a wide spectrum of symptoms of FS patients is associated with the type and location of the causative FOXG1 variants ^19,24^. This highlights the need for patient-specific FS mouse models, which carry FS-causing genetic variants, to understand FS pathophysiology and develop therapeutic interventions for FS patients. The existing Foxg1-null mouse lines ^2,9,25,26^ are inadequate as they have the complete deletion of the Foxg1 gene.

In this study, we report the first patient-specific FS mouse model that precisely mimics the predominant genetic variation of FS; Q84Pfs-Het mice that carry a heterozygous frameshift mutation in the Foxg1 gene. Remarkably, Q84Pfs-Het mice recapitulate not only FS-causing genotype but also diverse human FS symptoms from cellular to behavioral phenotypes, including uncoordinated movements and ASD-like behaviors. By studying the dysregulated genes in the cortex of Q84Pfs-Het mice, we identified the novel molecular pathways of FS in neurons and OL lineage. Together, our study with the newly established patient-specific FS mouse model provides important insights into the gene regulatory pathways of FOXG1 and the pathophysiology of FS. Further, our patient-specific FS mouse model creates the platform to develop FS therapeutic strategies.

## Results

### The coding region of FOXG1’s N-terminus is a mutation hotspot

**Figure 1:**
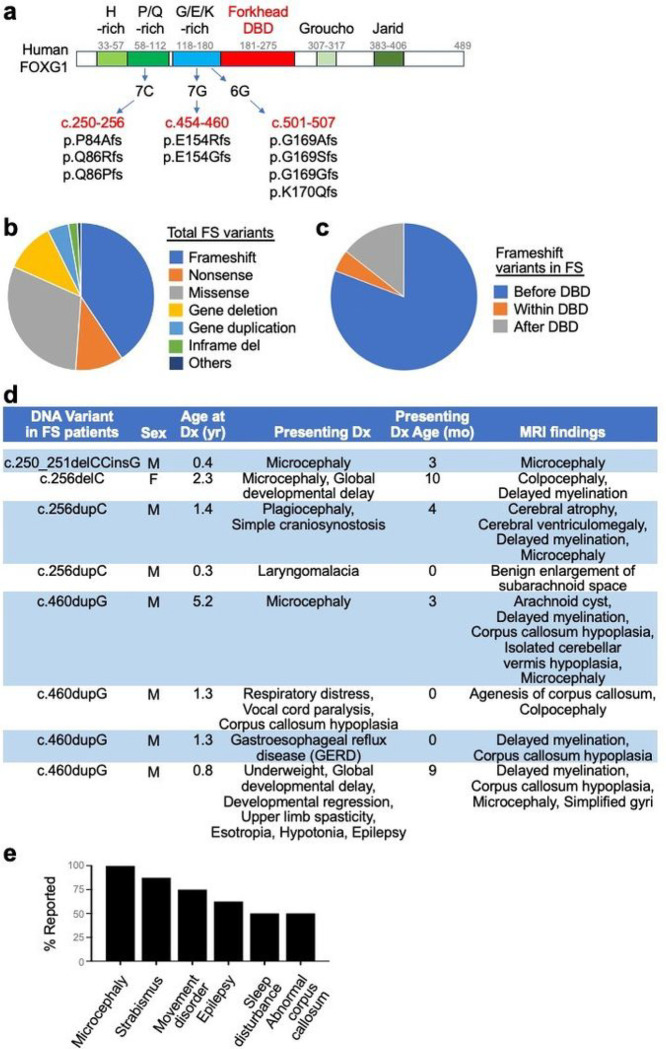
Frameshift mutations in the three mutation hot spots in the FOXG1 gene. **a,** Schematics of human FOXG1 protein and the three G (guanine) or C (cytosine)-repeat regions in the FOXG1 coding sequences. The three regions are mutation hot spots leading to FS. The frameshift type FOXG1 gene variants in these three hot spots were shown. These frameshift mutations occur in the N-terminal region of FOXG1. **b,c**, Pie charts showing the fraction of each mutation type that results in FS (**b**) and the fraction of frameshift mutations occurring before, within, or after the DNA-binding domain (DBD) in the FOXG1 protein (**c**). **d**, Clinical findings of eight FS patients with FOXG1 mutations at c.250–256 and c.454–460 positions. **e**, Frequency of different phenotypes among the eight FS patients in (**d**).

The human FOXG1 gene possesses three prominently high GC-rich regions in the coding sequences for the N-terminus of the FOXG1 protein, seven C (7C, c.250–256), seven G (7G, c.454–460), and six G (6G, c.501–506) (Fig. 1a). Correspondingly, FS-causing frameshift variants are over-represented in the N-terminal region of the FOXG1 protein prior to the DNA-binding Forkhead domain. In the FOXG1 Syndrome Research Registry, which enrolled 256 individuals with FS, frameshift variants are the most frequent, accounting for 40.6% of participants (104 out of 256 cases) (Fig. 1b). 81% of frameshift variants (84 out of 104 cases) occur before the DNA-binding domain (Fig. 1c). 64.4% of frameshift cases (67 out of 104 cases) were found in the 7C, 7G, and 6G regions, indicating that these C/G-rich regions are prone to the mutations that cause FS.

We reviewed the clinical data of eight individuals with FOXG1 variants at c.250–256 (7C) or c.454–460 (7G) positions (Fig. 1d,e). Over 50% of individuals with variants in these regions showed microcephaly, strabismus, movement disorder, hypotonia, epilepsy, sleep disturbance, and abnormalities of the corpus callosum agenesis (Fig. 1e). Five of eight patients also presented with delayed myelination detected via magnetic resonance imaging of the brain (Fig. 1d).

### Generation of mice carrying Q84Pfs allele

**Figure 2:**
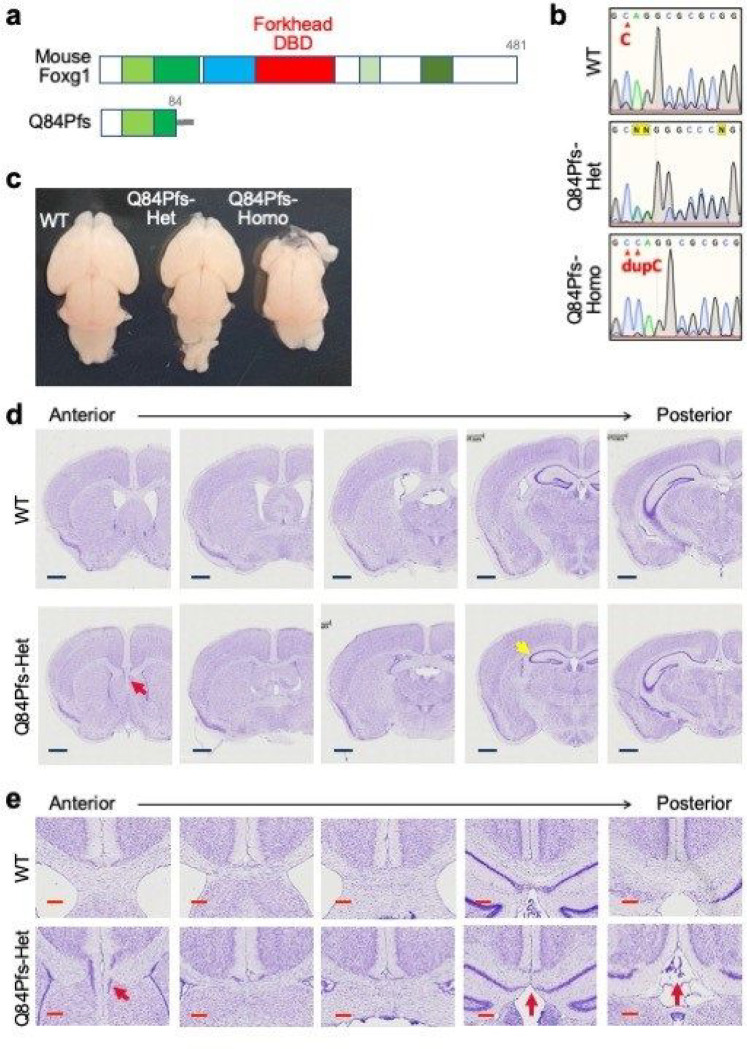
Anatomical deficits of the forebrains of Q84Pfs mice. **a,** Schematics of mouse Foxg1 full-length protein and Q84Pfs, a truncated form of Foxg1. **b,** Sanger sequencing results of the Foxg1 gene of wild-type (WT), Q84Pfs-Het, and Q84Pfs-Homo mice. **c,** The morphology of E18.5 WT, Q84Pfs-Het, and Q84Pfs-Homo brains. **d,** The representative images for Nissl stain of the serial coronal sections of WT and Q84Pfs-Het brains at P60. The anterior to posterior sections were as shown. **e,** The magnified views of the midline areas in (**d**). Scale bars, 1mm (**d**) or 200mm (**e**). The red and yellow arrows mark midline deficits and underdeveloped hippocampus, respectively, in Q84Pfs-Het brains.

To understand the pathophysiology of FS, we generated FS mouse model bearing a specific FS-causing FOXG1 variant. The knock-in mice carry c.250dupC (p.Q84Pfs*31, herein referred to as Q84Pfs), equivalent to human c.256dupC (p.Q86Pfs*35, referred to as Q86Pfs), representing one of the most prevalent variants in FS (Fig. 1a, 2a,b).

Foxg1^Q84Pfs/Q84Pfs^ homozygous (Q84Pfs-Homo) mice exhibited a marked loss of forebrain tissue and died soon after birth (Fig. 2c, Supplementary Fig. 1). They also showed craniofacial defects, such as a short frontonasal process, and underdeveloped eye morphology (Supplementary Fig. 1). In contrast, Foxg1^Q84Pfs/+^ heterozygous (Q84Pfs-Het) mice survived into adulthood.

### Structural brain deficits in Q84Pfs-Het mice

Compared to wild-type (WT) littermate controls, adult Q84Pfs-Het mice showed smaller brains, shortened corpus callosum, and profound septum defects (Fig. 2d,e). The hippocampus size was reduced, and the malformation of the dentate gyrus was notable (Fig. 2d). Thus, Q84Pfs-Het mice recapitulate the brain structural deficits in human FS with N-terminal frameshift variants (Fig. 1d,e).

### RNA-seq analysis identified the dysregulated gene sets in Q84Pfs-Het cortex

**Figure 3:**
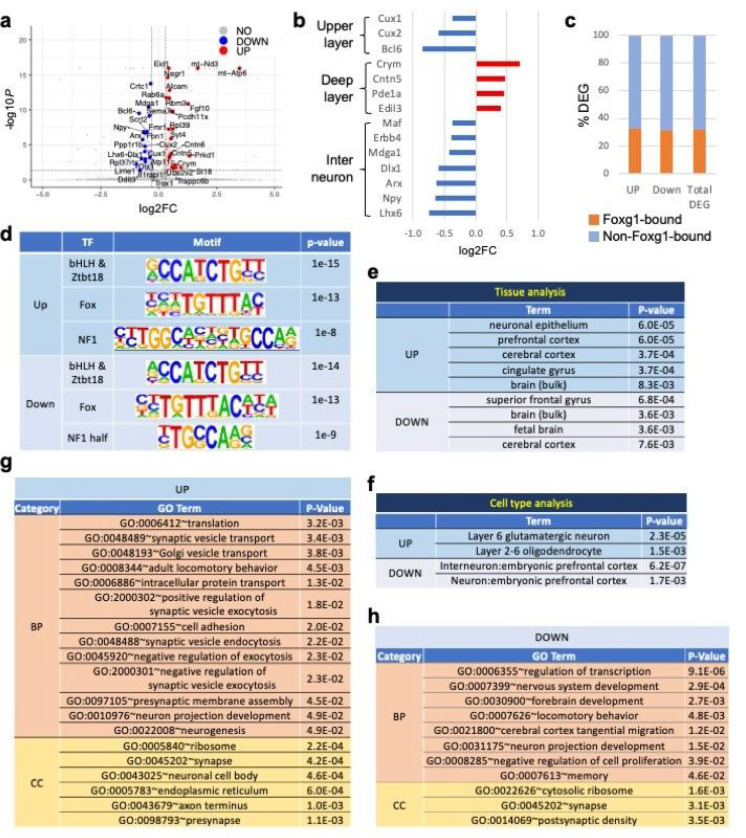
The dysregulated genes and pathways in Q84Pfs-Het cortex at P1. **a,** The differentially expressed genes (DEGs) in P1 cortices of Q84Pfs-Het mice as shown by the volcano plot. **b,** Cortical upper layer and interneuron genes were downregulated, whereas the deep layer genes were upregulated. **c,** The integration of DEGs of Q84Pfs-Het cortex and Foxg1 ChIP-seq data revealed the fraction of the DEGs that directly recruits Foxg1, as marked by orange. **d,** The motif analysis of Foxg1 ChIP-seq peaks associated with up- or down-regulated genes in Q84Pfs-Het cortex. TF, transcription factor. **e-h,** Gene set enrichment analysis (GSEA) of DEGs.

To identify the dysregulated genes contributing to the deficits of Q84Pfs-Het brains, we performed RNA-seq in P1 cortices of Q84Pfs-Het and littermate WT control mice and compared their gene expression patterns. 222 genes were significantly changed in the Q84Pfs-Het cortex relative to control WT cortex, representing differentially expressed genes (DEGs) (Fig. 3a, Supplementary Dataset). Interestingly, genes expressed in the cortical upper-layer projection neurons were downregulated, whereas deep-layer projection neuronal genes were upregulated (Fig. 3b), suggesting that the microcephaly of Q84Pfs-Het cortex is at least partly attributed to the reduction of the upper-layer, not deep-layer. Many genes involved in GABAergic interneuron development were also downregulated in Q84Pfs-Het cortex (Fig. 3b), indicating defects in the development of cortical interneurons.

Approximately 30% of DEGs in Q84Pfs-Het cortex were associated with Foxg1-bound ChIP-seq peaks in the developing cortex ^11^ regardless of up- or down-regulation (Fig. 3c), suggesting that Foxg1 regulates its direct target genes both negatively and positively. Consistently, the HOMER motif analysis^27^ revealed that the top two significantly enriched motifs in Foxg1-bound peaks annotated to either up- or down-regulated genes are the FOX motif and E-box. E-box serves as the binding site for basic helix-loop-helix (bHLH) and Zbtb18 (Rp58) transcription factors, both of which were shown to collaborate with Foxg1 11,28 (Fig. 3d). The NF1 site was also significantly associated with both up- and down-regulated Foxg1-target genes (Fig. 3d). These data suggest that a subset of dysregulated genes in Q84Pfs-Het mice are direct target genes of Foxg1 and that Foxg1 cooperates with ZBTB18, bHLH transcription factor, and NF1 in the developing cortex.

### The dysregulated pathways in Q84Pfs-Het cortex

To gain insights into molecular and cellular pathways leading to defects of Q84Pfs-Het mice, we performed the gene set enrichment analysis (GSEA) of DEGs using the Database for Annotation, Visualization and Integrated Discovery (DAVID) ^29^ and Enrichr ^30^.

In tissue analysis, DEGs were enriched in neuronal epitheliums, prefrontal cortex, cerebral cortex, cingulate gyrus, and superior frontal gyrus (Fig. 3e), consistent with the use of cortex for RNA-seq. In cell type analysis, the upregulated genes were significantly associated with cortical layer 6 glutamatergic neurons and, interestingly, oligodendrocytes, in which Foxg1 function remains unclear (Fig. 3f). The downregulated genes were most highly enriched for the “interneuron: embryonic prefrontal cortex” (Fig. 3f). Thus, our bioinformatic analyses indicate that the three major cell types in the neonatal cortex, excitatory projection neurons, inhibitory GABAergic interneurons, and the oligodendrocyte lineage, have a deficiency in Q84Pfs-Het mice.

Consistent with the known function of Foxg1 ^10–14^, DEGs of Q84Pfs-Het cortex were enriched for the genes controlling neuronal projection development, neuronal cell body, axon, and transcription regulations (Fig. 3g,h). Interestingly, the gene involved in the negative regulation of cell proliferation was downregulated in Q84Pfs-Het cortex (Fig. 3h), suggesting that progenitor proliferation may increase in Q84Pfs-Het cortex unlike the heterozygous mice carrying Foxg1-null allele (global Foxg1-Het)^2,5,8,9^.

Intriguingly, many synaptic genes were significantly dysregulated in Q84Pfs-Het cortex. The upregulated genes were enriched for biological process (BP) terms of synaptic vesicle transport, synaptic vesicle exocytosis and endocytosis, and presynaptic membrane assembly, and cellular component (CC) terms of synapse and presynapse (Fig. 3g,h). Further, the downregulated genes were also strongly associated with the CC terms of synapse and postsynaptic density (Fig. 3g,h). Our results underline the new role of Foxg1 in synapse development. It is also noteworthy that the upregulated and downregulated genes were more strongly associated with presynapse and postsynaptic density, respectively.

Remarkably, both up- and down-regulated genes were enriched for translation and ribosome (Fig. 3g,h), suggesting defects in translation in Q84Pfs-Het brains. Other enriched terms, such as locomotory behavior and memory, are related to FS symptoms, such as intellectual disability and hyperkinetic-dyskinetic movements ^19–23^.

Together, our results highlight the new role of Foxg1 in the oligodendrocyte development and synapse formation and function.

### Developmental defects of neural progenitors and excitatory and inhibitory neurons in Q84Pfs-Het cortex

**Figure 4:**
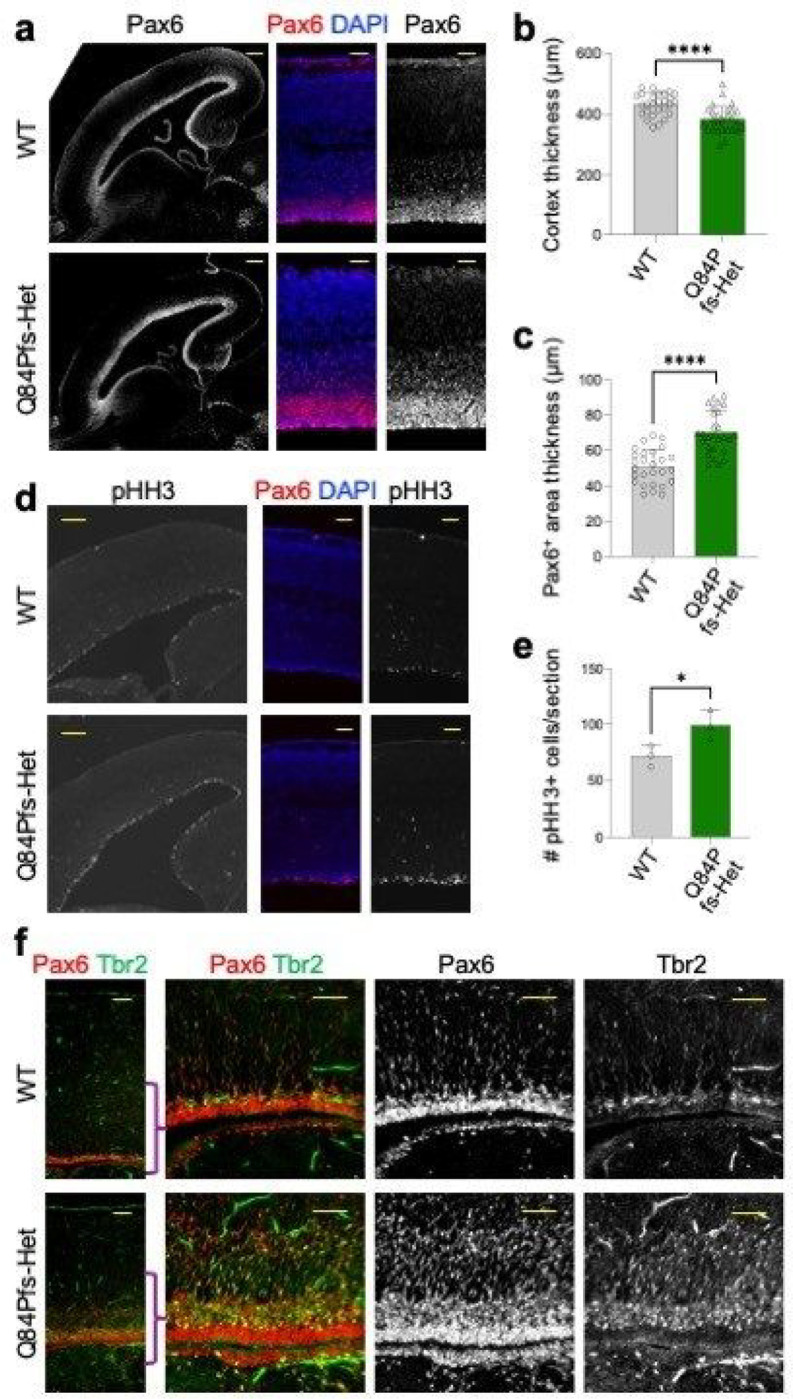
Q84Pfs-Het cortex showed increased Pax6^+^ and Tbr2^+^ neural progenitors. **a-f,** The immunostaining analyses of the sagittal sections of Q84Pfs-Het and WT cortices at E16 (**a-e**) and P1 (**f**). The quantification of cortex thickness (**b**), the thickness of Pax6^+^ progenitor areas (**c**), and the number of phosphorylated histone H3^+^ cells (**e**) of E16 cortices. Scale bars, 200mm (lower magnification images in **a, d**), 50mm (higher magnification images in **a, d**), or 100mm (**f**). Thickness in (**b,c**) was measured in three independent areas per section and three sections were analyzed in each mouse (n = 3 mice per group). The error bars (**b,c,e**) represent the standard deviation of the mean. *, *p*<0.05, ****, *p*<0.0001 in unpaired two-tailed test.

We next assessed if the dysregulated genes in Q84Pfs-Het brains led to cortical defects suggested by our GO analyses. Interestingly, although Q84Pfs-Het cortex was thinner than the WT cortex by E16 (Fig. 4a,b), Pax6^+^ neural progenitor cells (NPCs) and Tbr2^+^ intermediate progenitor cells (IPCs) increased in Q84Pfs-Het cortex at E16 and P0 (Fig. 4a–f). Consistently, phospho-histone H3^+^ proliferating cells in the ventricular zone were also significantly increased in Q84Pfs-Het cortex (Fig. 4a–f). Combined with the downregulation of genes for negative regulation of cell proliferation (Fig. 3h), these results indicate that progenitor proliferation was augmented in Q84Pfs-Het mice in contrast to the reduced progenitor proliferation in global Foxg1-Het mice ^2,5,8,9^.

**Figure 5:**
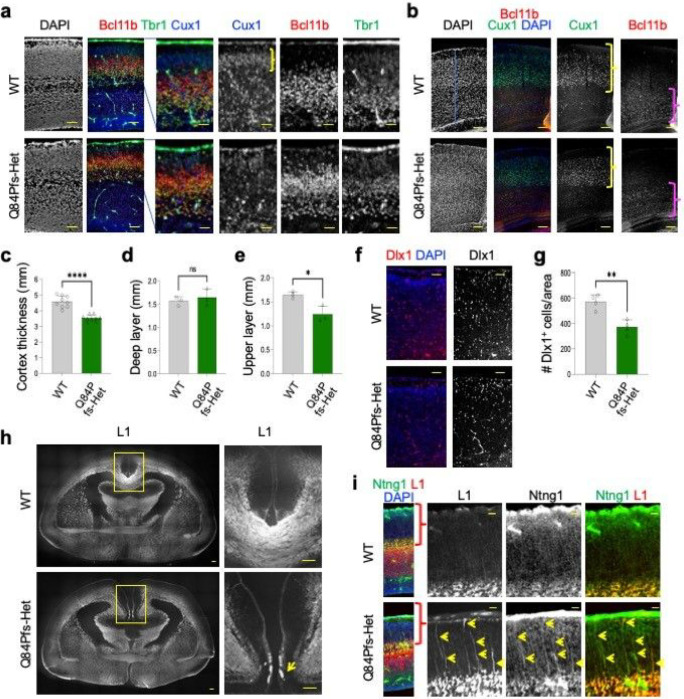
Q84Pfs-Het mice showed a reduction of cortical thickness, upper cortical layer, and cortical interneurons, and axon misprojections in the cortex. **a-i,** The immunostaining analyses of Q84Pfs-Het and WT brains at E16 (**a,i**), P30 (**b-e**), P1 (**f, g**), and P0 (**h**). The Cux1^+^ upper layer was marked by yellow brackets (**a,b**), and the deep layer was marked by magenta brackets (**b**). **c-e**, The quantification of cortex thickness (**c**), the thickness of the deep layers 5/6 (d) and the thickness of the Cux1^+^ upper layer (**e**) in three independent areas per mouse (n = 3 mice per group). **g,** The number of Dlx1^+^ cortical interneurons in two independent sections per mouse (n = 2 mice per group). **h,** The immunostaining with L1 axonal marker revealed the corpus callosum agenesis in Q84Pfs-Het brains. The yellow arrow indicates the Probst bundle at the midline. i, The misprojection of the bundle of L1/Ntng1 (NetrinG1)-double positive axons (yellow arrows) in Q84Pfs-Het cortex. Scale bars, 200mm (lower magnification images in **a**), 100mm (higher magnification images in **a**), 500mm (**b,f**), 1mm (**h**), and 200mm (**i**). **c-e, g**, The error bars represent the standard deviation of the mean. *, *p*<0.05, **, *p*<0.01, ***, *p*<0.001, ****, *p*<0.0001; ns, non-significant in unpaired two-tailed test.

The production of Cux1^+^ upper-layer neurons was delayed in E16 Q84Pfs-Het cortex, as shown by a lack of Cux1^+^ neurons in the superficial area of the cortex (Fig. 5a). Cux1^+^ upper-layer neurons remained significantly reduced in P30 Q84Pfs-Het cortex (Fig. 5b,e), indicating that the delayed upper-layer neuron generation is not compensated at the later stages. The deep-layer neurons showed a trend of increase in number, but the thickness of the deep-layer did not significantly increase (Fig. 5a,b,d). Combined with reduced cortex thickness (Fig. 5. a–c), these results show that mainly the reduction of the upper-layer accounts for the thinner cortex.

Dlx1^+^ GABAergic interneurons were substantially decreased in Q84Pfs-Het cortex (Fig. 5f,g), consistent with downregulation of genes involved in cerebral cortex tangential migration (i.e., GABAergic interneuron migration deficits) (Fig. 3f,h).

Q84Pfs-Het mice displayed two prominent axon projection deficits in the cortex, matching the dysregulation of neuron projection development genes (Fig. 3g,h). First, a substantial fraction of callosal axons was stalled at the midline, forming the Probst bundle (Fig. 5h). Second, Q84Pfs-Het showed misprojected L1^+^ axon bundles crossing the cortical plate toward the superficial area. These misrouted L1^+^ axons expressed Ntng1 (netrin G1) (Fig. 5i), indicating the defects of thalamocortical axon guidance in Q84Pfs-Het cortex.

Together, Q84Pfs-Het cortex exhibited deficits in progenitor proliferation, axon projection, layer formation, and interneuron migration.

### Myelination defects in Q84Pfs-Het cortex

**Figure 6:**
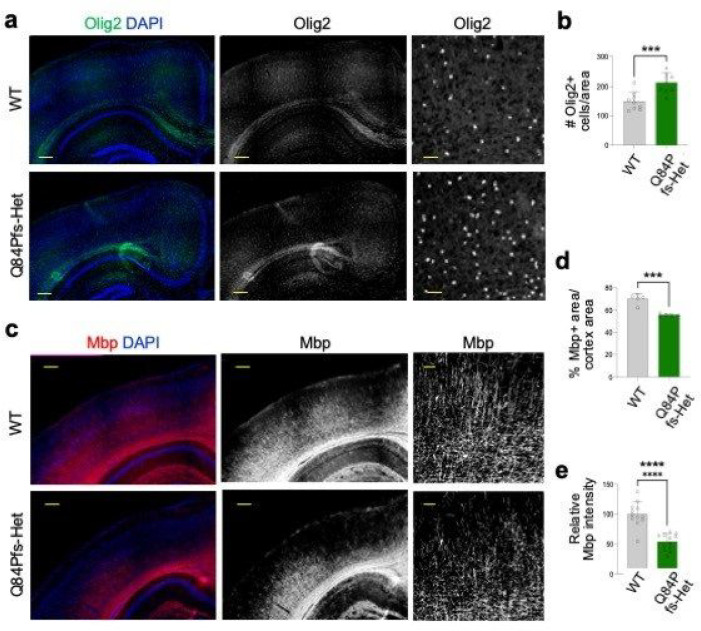
Q84Pfs-Het mice showed myelination deficits. **a-f,** The immunostaining analyses of Q84Pfs-Het and WT brains at P30. The quantification of the number of Olig2^+^ oligodendrocyte lineage cells (**b**; in three independent areas per mouse, n = 3 mice per group), the percentage of strong Mbp^+^ myelinated axon areas per the total cortex area (**d**; in two independent areas per mouse, n = 3 mice per group), and the relative Mbp intensity in the cortex (**e**; in three independent areas per mouse, n = 3 mice per group). Scale bars, 1mm (lower magnification images in **a,c**), 100mm (higher magnification images in **a**), and 200mm (higher magnification images in **c**). **b,d,e,** The error bars represent the standard deviation of the mean. **, *p*<0.01, ***, *p*<0.001, ****, *p*<0.0001 in unpaired two-tailed test.

Although delayed myelination is one of the most common features of FS brains (Fig. 1d), the role of Foxg1 in the oligodendrocyte lineage development remains unclear. To test if the FS mouse model recapitulates this prominent white matter phenotype in humans, we monitored oligodendrocyte differentiation and myelination in Q84Pfs-Het mice at P30, the active myelination phase. Interestingly, Olig2^+^ oligodendrocyte lineage cells, many of which include oligodendrocyte progenitor cells (OPCs), significantly increased in Q84Pfs-Het cortex (Fig. 6a,b), consistent with the association of upregulated genes with oligodendrocyte (Fig. 3f). Despite of the increased Olig2^+^ cells, Mbp^+^ myelinated area was substantially reduced in Q84Pfs-Het brains (Fig. 6c,d). Moreover, Q84Pfs-Het brains exhibited a marked reduction of the arborization and complexity of myelination patterns (Fig. 6c,e). Our results indicate that the oligodendrocyte development process was disrupted, and the myelination steps, including the microstructural organization of myelination, was impaired in Q84Pfs-Het brains, providing the pathological mechanisms underlying white matter deficiency in FS.

### Dysregulated genes in Q84Pfs-Het cortex were associated with motor dysfunction and autistic-like behaviors

**Figure 7:**
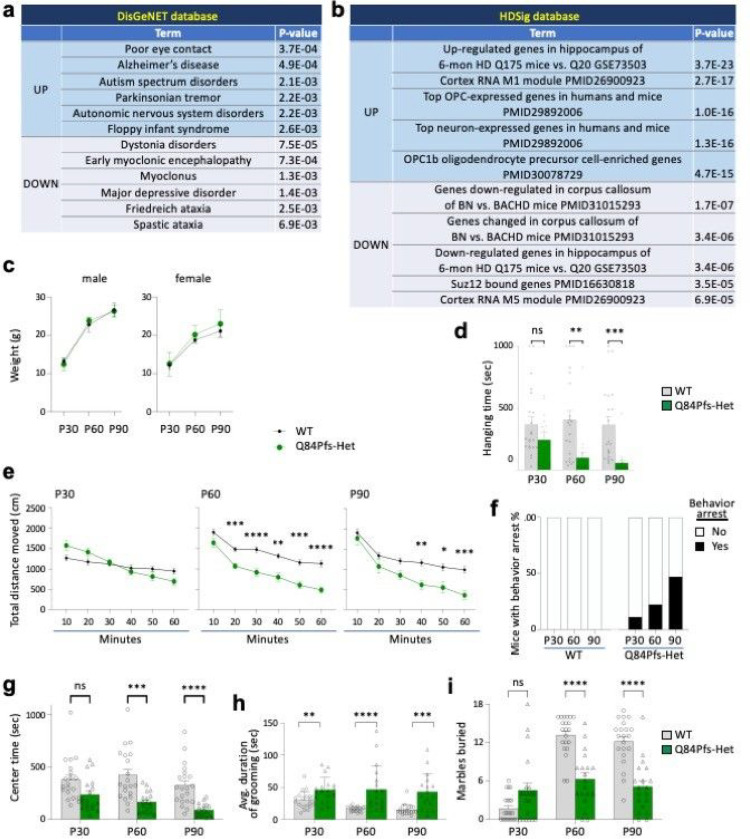
Q84Pfs-Het mice showed movement deficits, autism-like repetitive behaviors, and prolonged behavior arrest. **a,b,** Gene set enrichment analysis (GSEA) revealed the significant association between the DEGs in Q84Pfs-Het cortex and human disorders, such as autism (**a**), and dysregulated genes in Huntington’s disease mouse models and OPC genes (**b**). **c,** WT and Q84Pfs-Het mice showed no significant difference in body weights (WT 12 male, 9 female; Q84Pfs-Het 9 male, 10 female). **d,**Q84Pfs-Het mice showed a reduced hanging time. **e,** Q84Pfs-Het mice showed reduced travel distance at P60 and P90. **f,** Some Q84Pfs-Het mice exhibited prolonged behavior arrest, which is defined by paused locomotion for longer than 3 min at one episode during the open field test. In contrast, no WT mice showed prolonged behavior arrest. The black bars indicate % of mice showing behavior arrest among all tested mice. **g,** Q84Pfs-Het mice showed a reduced center time. **h**, Q84Pfs-Het mice exhibited significantly increased self-grooming behavior relative to WT, as measured by the average duration of an individual grooming event. **i,** Q84Pfs-Het mice buried significantly fewer marbles than WT mice at P60 and P90 but showed a tendency of burying more marbles at P30. *c-i,* Error bars, SEM. *, *p*< 0.05; **, *p*<0.01; ***, *p*<0.001; ****, *p*<0.0001; ns, not significant in the two-way ANOVA test (**c,d,e,g,i**) or the Mann-Whitney Test (**h**). Each circle and triangle correspond to one animal. **d,i,** For P30, WT n = 22 (12 male, 10 female), Q84Pfs-Het n= 20 (10 male, 10 female); for P60 and P90, WT n = 21 (12 male, 9 female), Q84Pfs-Het n = 19 (9 male, 10 female). **e-h,** For P30 and P60, WT n = 20 (12 male, 8 female), Q84Pfs-Het n = 18 (8 male, 10 female); for P90 in **e,g,** WT n = 21 (12 male, 9 female), Q84Pfs-Het n =18 (8 male, 10 female) and for P90 in **f,h,** WT n = 21 (12 male, 9 female), Q84Pfs-Het n = 17 (7 male, 10 female).

To ask if the dysregulated genes in the Q84Pfs-Het cortex are connected to specific human conditions, we performed GSEA with the DisGeNET database, which contains collections of genes and variants associated with human diseases ^31^ (Fig. 7a). The upregulated genes were significantly associated with impaired social interactions, Alzheimer’s disease, ASD, Parkinson’s disease, and autonomic nervous system disorders (Fig. 7a). The downregulated genes were enriched for dystonia, myoclonic encephalopathy, myoclonus, and ataxia, as well as major depressive disorder. Together, Q84Pfs-Het transcriptome changes strongly linked to impairments in movement, social interactions, and autonomic nervous system function in Q84Pfs-Het mice.

Given that both FS and Huntington’s disease (HD) exhibit many neurological symptoms, including abnormal involuntary movements ^22,32^, we compared the dysregulated genes between Q84Pfs mice and HD using the database of HD molecular signatures (HDSigDB, hdinhd.org) (Fig. 7b). This analysis revealed the striking resemblance between Q84Pfs-Het mice and HD mouse models ^33,34^. The high-ranking categories that resemble the up- and down-regulated genes in Q84Pfs-Het mice were the up- and down-regulated genes in the hippocampus of the HD mouse model Q175 ^33^, respectively (Fig. 7b). Notably, the two categories among the top five gene sets for the upregulated genes were oligodendrocyte progenitor cell (OPC) genes (Fig. 7b), consistent with our finding that Q84Pfs-Het cortex showed the increased Olig2^+^ oligodendrocyte lineage cells (Fig. 6a,b).

Collectively, the transcriptome changes in Q84Pfs-Het cortex are closely connected to the clinical features of FS, such as movement disorders, autism-like behaviors, and social impairment.

### Q84Pfs-Het mice showed movement deficits, repetitive behaviors, and prolonged behavior arrest

To investigate whether Q84Pfs-Het mice exhibit behavior phenotypes corresponding to the above molecular and cellular changes, we performed behavioral assessment tests at adolescence stage P30 and adult stages P60 and P90.

There was no significant difference in body weights of Q84Pfs-Het and WT mice at these stages (Fig. 7c). In the wire hang test that evaluates the motor function and muscle strength, Q84Pfs-Het mice showed significantly reduced hang time, which worsened as mice aged (Fig. 7d). In the open field test that monitors locomotor activity and exploratory behaviors, Q84Pfs-Het mice moved significantly less than WT mice at P60 and P90 (Fig. 7e). At P30, Q84Pfs-Het mice showed a tendency for travel distance reduction over time compared to WT mice, but the total travel distance did not differ significantly (Fig. 7e). These results indicate deficits in muscle strength and movement in Q84Pfs-Het mice.

Q84Pfs-Het mice exhibited a strikingly extended behavior arrest, defined by paused locomotion for longer than 3 min at one episode during free exploration of the arena (Fig. 7f, Supplementary Fig. 2 and videos). Throughout these episodes of remarkably pronounced stops, Q84Pfs-Het mice showed a high degree of postural control, typically positioning their bodies facing the center of the arena, and no apparent overt movements, such as visual survey, rearing, or digging. The proportion of Q84Pfs-Het mice that exhibited the extended behavior arrest was 11%, 22%, and 47% at P30, P60, and P90, respectively, whereas WT mice did not show behavior arrest without purposeful movements under the same condition (Fig. 7f). Notably, Q84Pfs-Het mice without prolonged behavior arrests still showed a decreased travel distance, suggesting that the extended arrest is not the only contributing factor to movement reduction.

Next, we assessed ASD-related behaviors. The center time in the open field test was markedly decreased as Q84Pfs-Het mice aged from P30 to P90, relative to WT mice (Fig. 7g), indicating increased anxiety levels. Q84Pfs-Het mice exhibited a significant increase in repetitive grooming behavior at all three stages, with a tendency for bigger differences from WT mice as they age (Fig. 7h). These results suggest heightened repetitive behavior in Q84Pfs-Het mice. Intriguingly, in the marble-burying test, Q84Pfs-Het mice buried more marbles at P30 but fewer marbles at the adult stages than WT mice at P60 and P90 (Fig. 7i), suggesting age-dependent changes in the marble burying behavior.

Together, our data uncovered that Q84Pfs-Het mice have motor coordination defects, increased anxiety levels, remarkably prolonged behavior arrest, and repetitive behaviors, all observed in FS ^19–23^ and strongly linked to molecular and cellular changes of Q84Pfs-Het brains.

## Discussion

A majority of FS patients possess mutations within the FOXG1 gene coding region, which are likely to produce faulty Foxg1 protein products that impact disease mechanisms and progression. Therefore, the currently available Foxg1-null mouse lines are inadequate to investigate FS pathophysiology because they delete the entire Foxg1 coding region ^2,9,25,35,36^. Here, we established Q84Pfs-Het mice as the first patient-specific FS mouse model that accurately replicates human genetic conditions of FS. Remarkably, the heterozygous mice carrying only a single allele of Q84Pfs variant, without creating the homozygous conditions, recapitulated a wide range of human FS symptoms, such as brain structural deficits and ASD-like behaviors. Using this new FS mouse model, we uncovered molecular and cellular changes, such as oligodendrocyte lineage deficiency and dysregulation of synaptic genes, leading to the key hallmarks of FS, such as delayed myelination, movement deficits, and ASD-like behaviors.

A tight genetic and phenotypic resemblance between Q84Pfs-Het mice and human FS patients provides clear advantages in understanding the pathophysiology of neurodevelopmental disorders and developing therapeutics, compared to many ASD mouse models, in which the ASD genes are eliminated, unlike the corresponding human conditions in which ASD genes are often present as heterozygous conditions or are epigenetically silenced ^37^. First, it allowed us to identify dysregulated genes in FS brains, which are likely to lead to cellular and structural deficits and further behavioral outcomes. Second, our studies uncovered new mouse phenotypes highly correlated with human FS symptoms, such as deficiency in oligodendrocyte development and myelination, increased repetitive behavior and anxiety levels, and extensive behavior arrest. Thus, Q84Pfs-Het mice will serve as an ideal mouse model to identify the pathogenic mechanism for these mouse phenotypes and human symptoms. Third, Q84Pfs-Het mouse model enables investigation of the role of the truncated form of Foxg1 resulting from frameshift mutations in FS. Foxg1 mRNA level did not decrease in Q84Pfs-Het cortex (Supplementary dataset), suggesting that Foxg1 mRNA from Q84Pfs-Het allele did not undergo non-sense mediated decay. These data also suggest that Q84Pfs allele in mice (Q86Pfs allele in humans) expresses the N-terminal fragment of the Foxg1 protein. Notably, the Foxg1 fragment produced from mouse Q84Pfs and human Q86Pfs alleles would have interesting amino acid signatures of histidine, proline, and glutamine repeats (Fig. 1a). In this regard, it is noteworthy that Q84Pfs-Het mice show phenotypes distinct from the existing global Foxg1-Het mice. Q84Pfs-Het mice show increased NPC proliferation while global Foxg1-Het mice exhibited reduced NPC proliferation ^38^. It will be interesting to examine the molecular basis of this stark difference. Fourth, the Foxg1 heterozygous condition of Q84Pfs-Het mice enables us to evaluate the therapeutics that will not be assessable in homozygous mouse models, such as an approach to enhance Foxg1 expression from the WT Foxg1 allele, resulting in a boost of functional Foxg1 proteins.

Our parallel studies of Q84Pfs-Het mice at molecular and behavioral levels provided important insights into FS pathogenesis. Q84Pfs-Het cortex showed the gene expression changes that predict various movement deficits and ASD-like behaviors. Consistently, adult Q84Pfs-Het mice exhibited the corresponding behavioral characteristics. This close matching of gene and behavior signatures pointed to the genes whose misexpression leads to movement deficits and ASD-like behaviors in Q84Pfs-Het mice. Fmr1, Nalcn, Il1rapl1, and Slc16a2, which are aberrantly upregulated in Q84Pfs-Het brains, are likely to be involved in intellectual disability, social impairment, and autism-like behaviors, given that these genes are associated with neurodevelopmental disorders with the described signature behaviors ^39–42^. A subset of FS symptoms, such as dystonia, myoclonus, and ataxia, may be attributed to the downregulation of PLEKHG2, DST, ARX, AAAS, KCTD17, PRDM8, and CRTC1 in FS brains as these genes are downregulated in Q84Pfs-Het brains and play a role in these movement and muscle coordination ^43–49^. The remarkable similarity of dysregulated genes between Q84Pfs-Het and HD mouse models suggests that the shared gene expression changes are responsible for common symptoms between FS and HD, such as involuntary jerky and writhing movement. Our studies also revealed that the genes involved in synaptic vesicle exocytosis, endocytosis, and synapse organization and membrane assembly are dysregulated in Q84Pfs-Het cortex, strongly implying synapse deficiency as a mechanism driving FS symptoms, such as prolonged behavior arrests and cognition impairments. Of note, the prolonged behavior arrest similar to the behavior of Q84Pfs-Het mice (Fig. 7f) has been described for seizures, like focal behavior arrest seizures (aka, focal impaired awareness seizures, hypokinetic seizure, localized seizure with behavior arrest, or complex partial seizures) ^50–53^. As this type of seizure has been observed in FS ^54^, it will be interesting to perform electroencephalogram (EEG) recording paired with behavioral assessment in Q84Pfs-Het mice and examine if behavior arrest is correlated with specific types of EEG activities. Given the symptomatic similarity between FS and other neurological disorders, the pathogenic mechanism studies in Q84Pfs-Het mice will provide significant insights into a broader range of neurodevelopmental disorders, including ASD.

Q84Pfs-Het brains faithfully captured the structural deficits of the cortex, a brain region mainly derived from the dorsal telencephalon, in FS patients. The future investigation can determine if the ventral telencephalon-derived brain deficits, such as the striatum, match between Q84Pfs-Het mice and FS patients and if the structural changes and gene dysregulation in the striatum correlate with the behavior of Q84Pfs-Het mice. As the striatum is strongly involved in motor control and compulsive and anxiety behaviors and is one of the main areas affected in HD and Parkinson’s diseases ^32^, the striatal circuits may play a role in a subset of behavioral phenotypes of Q84Pfs-Het mice.

The myelination deficits in Q84Pfs-Het mice suggest a key role of Foxg1 in oligodendrocyte development. In Q84Pfs-Het brains, Olig2^+^ oligodendrocyte lineage cells increase, despite disrupted myelination (Fig. 6), suggesting Foxg1 functions at multiple points of oligodendrocyte development. Overexpression of Foxg1 inhibits the generation of OPC from neural progenitors in vitro ^55^ and Foxg1 deletion specifically in adult neural stem cells decreases OPC proliferation and facilitates remyelination in Cuprizone-induced demyelination conditions ^56^. Combined with these reports, our results raise the possibility that Foxg1 haploinsufficiency triggers the overproduction of Olig2^+^ OPCs from neural progenitors but inhibits differentiation and maturation of oligodendrocytes, thereby resulting in accumulated OPCs and reduced myelination. Future studies are needed to test this possibility. The myelination deficiency likely contributes to various FS symptoms, such as dystonia. Further investigation into dysmyelination of Q84Pfs-Het brains will uncover not only pathogenic mechanisms of delayed myelination, a hallmark of FS brains, but also key regulatory steps in OL development. Considering that proper myelination of nerves is essential for movement and diverse brain functions and that myelination primarily begins after birth and continues into young adulthood in humans ^57^, dysmyelination could be an excellent therapeutic target for FS.

Foxg1 plays cell type-specific and developmental timing-dependent roles in forebrain development, at least via the two mechanisms. First, the dynamic expression of Foxg1 plays a key role. The downregulation of Foxg1 during neurogenesis is crucial for the cell cycle exit and differentiation of neural progenitors ^8,9,58,59^. After neuronal differentiation, Foxg1 expression is induced in postmitotic neurons, in which Foxg1 promotes neuronal morphological transition and migration ^10,11^. Second, Foxg1 partners with other transcription factors in a cell context-specific manner in selecting target genes. Our motif analysis of Foxg1-bound genomic loci of DEGs identified E-box, the binding sites for Foxg1’s partner transcription factors Zbtb18 and Neurod1 ^11,28^, along with the Fox motif. Our data integrating RNAseq and ChIPseq datasets (Fig. 3d) also suggest that NF1 collaborates with Foxg1 in Foxg1-mediated transcriptional regulation in the cortex. Notably, NF1 is a family of closely related transcription factors, NF1A, NF1B, NF1C and NF1X, and in particular NFIA and NFIB have been found to be involved in haploinsufficiency syndromes that share some features with the FS, such as corpus callosum agenesis and intellectual disability^60–62^.

Collectively, our study established Q84Pfs-Het mice as the FS mouse model that will expedite our understanding of the pathophysiology of FS and serve as an essential platform for therapeutic development. Importantly, our study uncovered new cellular and behavioral phenotypes that are linked to Foxg1-dependent gene expression changes in Q84Pfs-Het mice, providing key insights into Foxg1 function and FS etiology.

## Methods

### FOXG1 Syndrome Research Registry & Ciitizen Databank

The International FOXG1 Syndrome Research Registry is an online platform that collects information from caregivers of children and adults with FS, including results of genetic testing, clinical phenotypes, and developmental outcomes [https://foxg1.beneufit.com/login.php].

Ciitizen^®^, a wholly owned subsidiary of Invitae Corporation, is a patient-centric real world data platform that transforms medical records into structured datasets that describe clinical phenotypes and medical interventions [https://www.medrxiv.org/content/10.1101/2023.03.02.23286645v1]. In partnership with the FOXG1 Research Foundation, > 120 individuals with a FOXG1 variant have enrolled in Ciitizen. In brief, the platform supports medical records that are collected on behalf of participants through the Health Insurance Portability and Accountability Act (HIPAA). Following record collection, each medical document is systematically reviewed for relevant data variables, including presenting diagnoses and diagnostic imaging results. Genetic variants were extracted from clinical genetic test reports; loss-of-function variants in FS, including frameshift variants, are known to be pathogenic. To support harmonization of disparate data sources, extracted phenotypic data were mapped to SNOMED CT, an internationally recognized terminology (US Edition, version 2022_03_01) [www.snomed.org]. The resulting data elements were stored securely in HIPAA-compliant, controlled access, indexed database.

Caregivers and/or legal guardians of study participants provided broad consent to share de-identified data for research. The generation and subsequent analysis of participant data used in this study received determinations of exemption through a central institutional review board, Pearl IRB. Reasonable requests for data access can be made to cii-research@invitae.com.

### Animals

Q84Pfs mutant mice were generated with the CRISPR/Cas9 gene editing system using guide RNA (CCCAGCAGCCGACGACGACA) and single stranded oligonucleotides (ssODN) containing the mutation c.250dupC to introduce the Q84Pfs allele and the restriction enzyme site (HindIII) for genotyping (T CCA CCG CCG CCC CAG CAG CAG CAG CAG CAG CCG CCC CCG GCC CCG CAG CCC CCG C CAG GCG CGC GGC GCC CCA GCA GCC GAC GAC GAC AAG CTT CCC CAG CCG CTC CTG CTC CCG CCC TCC ACC). Potential founder mice were genotyped by Sanger sequencing and restriction enzyme digestion for PCR product surrounding the target site. The targeted founder mice from CRISPR/Cas9 microinjection were backcrossed to C57BL/6J mice for at least five generations to eliminate potential off-target mutations before any experiments.

### Behavioral tests

Mice were maintained on a 12 h light/dark cycle, and all behavioral tests were performed during the light phase. All animals were allowed to habituate in the behavior testing room (with attenuated light and sound) for at least 30 min before the tests. All arenas and objects used in behavioral analysis were cleaned with Alconox and then distilled water between test sessions.

Open field test: The open field test was utilized to examine locomotor activity and to monitor anxiety-like behaviors as well as repetitive behaviors such as jumping and self-grooming. Mice were placed into a Plexiglas chamber with transparent walls and white floor (40cm W X 40cm D X 30cm H) and then allowed to move freely for 60min. All activity was monitored by SuperFlex Open Field system (Omnitech Electronics) and the total moved distance and the time spent in the center (20 cm X 20 cm within the center of the floor) were collected and analyzed by Fusion software (AccuScan Instruments Incorporated). Grooming: Grooming was assessed manually using video recordings from the open field test. Each mouse was monitored for 60 minutes. For a grooming event to count, the mouse had to groom for at least 2 seconds. If the mouse stopped grooming, but resumed in 5 seconds or less, it was counted as the same grooming event. If the mouse moved to a new location in the cage and continued grooming, even if it resumed in 5 seconds or less, it was counted as a new grooming event. Prolonged behavior arrest: During the open field test, prolonged behavior arrest was marked whenever a mouse remained sitting in one location in the cage, largely unmoving, for at least 3 minutes. During this time, the mice did not appear to perform any purposeful movement, such as grooming, rearing, digging, or other exploratory behavior. Wire hanging test: Mice were placed inside a basket with wire grids on the bottom, and the basket was slowly inverted to allow the animal to hang from the grid. All sessions were video-recorded, and the hanging time was analyzed manually. The hanging time was defined from the time the basket was inverted to the time that the mouse fell off the wire grid. Mice were tested on three consecutive trials with at least 5 min intervals between trials. All trials ended upon reaching 1000 seconds. Marble burying: Mice were gently placed in the periphery of testing cages (arena size: 30 cm × 20 cm × 13 cm, corncob bedding depth: 5 cm) containing 18 glass marbles (evenly spaced with three rows of six marbles on the top of bedding), and then the cages were covered with the transparent top (height 13 cm). Mice were carefully removed from the cages, and the number of marbles buried was recorded at the end of the 30 min exploration period. Marbles were counted as buried when they have covered at least 2/3 depth with bedding.

### Brain preparation and immunohistochemical analysis

Dissected brains were fixed in 4% paraformaldehyde in phosphate-buffered saline (PBS) at 4°C overnight, equilibrated in 30% sucrose, and embedded in Tissue Freezing Medium^™^ (Electron Microscopy Sciences) for frozen sectioning for 18 μm brain sections using cryostat (CM1950, Leica). Position matched sections were then stained following the standard immunohistochemical method. Sections were incubated with the primary antibody and the secondary antibodies conjugated to fluorophores (Jackson ImmunoResearch), and then counterstained with DAPI to reveal nuclei. Images were collected through DM6 fluorescence microscope (analyzed by Leica Software LasX) or Eclipse Ti2 confocal microscope (analyzed by Nikon Software). The primary antibodies included rabbit anti-Olig2 (Millipore AB15328, 1:1000), rat anti-L1 (NCAM) (Millipore MAB5272, 1:1000), rat anti-Ctip2 (Abcam AB18465, 1:1000), rat anti-MBP (Millipore MAB386, 1:500), rabbit anti-Cux1 (Santacruz sc-13024, 1:500), chicken anti-Tbr1 (Millipore AB2261, 1:1000), rabbit anti-Dlx1 (Chemicon AB5724, 1:1000), and goat anti-Netrin-G1a (R&D AF1166, 1:500) antibodies. The secondary antibodies from Jackson ImmunoResearch Laboratories included donkey anti-rat Cy3 (Cat.712–165-153, 1:500), donkey anti-rabbit Alexa Fluor 647 (Cat.711–605-152, 1:500), donkey anti-rabbit Alexa Fluor 488 (Cat.711–545-152, 1:500), donkey anti-chicken Alexa Fluor 488 (Cat.103–545-155, 1:500) and goat anti-rat Alexa Fluor 594 (Cat.112–585-167, 1:500) antibodies.

### Nissl staining

Slides with cryosectioned frozen sections were incubated in ethanol/chloroform mixture, mixed 1:1 volumetrically, at RT overnight. Incubated slides went through rehydration steps in three steps, 100% ethanol, 95% ethanol and then distilled water for 10 minutes each. After rehydration, slides were stained in warm 0.2% cresyl violet solution for 12 min, followed by brief washing and then incubating in 95% ethanol, 100% ethanol and xylene for 20 minutes each for differentiation, dehydration, and clearing, respectively. Coverslip was mounted with Permount^™^ Mounting Medium (Fisher Scientific). Images from 18μm thick stained specimen were acquired by Axio Scan.Z1 (Zeiss) under bright field with 10X objective.

### RNA-seq data analyses

FastQC was used to check the quality of the raw RNA-seq data. Clean reads were aligned to the mouse reference genome (mm10) using STAR (v2.6.1d) ^63^ with default parameters and the gene expression levels were quantified using RSEM (v1.3.3) ^64^. The DEGs were identified using EBseq ^65^ based on the expected counts. The absolute value of log2 fold change of 0.3 and false discovery rate (FDR) of 0.05 were set as criteria to determine if the expression level of a gene was significantly changed. In this manner, 222 DEGs including 118 upregulated and 104 downregulated DEGs were identified in total. We subsequently carried out GSEA using DAVID and Enrichr on the upregulated DEGs and the downregulated DEGs, separately.

### Statistical analyses

The data were analyzed using GraphPad 9.5 (Prism, San Diego, CA). Student T-test were run using genotype as between-subject variables. A repeated-measures T-test was used to analyze activity in Olig2 positive cell counting, cortex thickness measuring, Pax6 positive area measuring, layer size measuring and DG length/angle measuring. Statistical significance was assessed using the Mann-Whitney or ANOVA test, as indicated in the figure legends, and was represented on the graphs as: ****p < 0.0001; ***p < 0.001; **p < 0.01; *p < 0.05; ns, not significant. All bars and error bars were specified in the figure legends.

## References

[1] Tao W. & Lai E. Telencephalon-restricted expression of BF-1, a new member of the HNF-3/fork head gene family, in the developing rat brain. Neuron 8, 957–966 (1992). 10.1016/0896-6273(92)90210-5 1350202

[2] Xuan S. Winged helix transcription factor BF-1 is essential for the development of the cerebral hemispheres. Neuron 14, 1141–1152 (1995). 7605629 10.1016/0896-6273(95)90262-7

[3] Cadwell C. R., Bhaduri A., Mostajo-Radji M. A., Keefe M. G. & Nowakowski T. J. Development and Arealization of the Cerebral Cortex. Neuron 103, 980–1004 (2019). 10.1016/j.neuron.2019.07.009 31557462 PMC9245854

[4] Lim L., Mi D., Llorca A. & Marín O. Development and Functional Diversification of Cortical Interneurons. Neuron 100, 294–313 (2018). 10.1016/j.neuron.2018.10.009 30359598 PMC6290988

[5] Martynoga B., Morrison H., Price D. J. & Mason J. O. Foxg1 is required for specification of ventral telencephalon and region-specific regulation of dorsal telencephalic precursor proliferation and apoptosis. Developmental biology 283, 113–127 (2005). 10.1016/j.ydbio.2005.04.005 15893304

[6] Manuel M. N. The transcription factor Foxg1 regulates telencephalic progenitor proliferation cell autonomously, in part by controlling Pax6 expression levels. Neural Dev 6, 9 (2011). 10.1186/1749-8104-6-9 21418559 PMC3068069

[7] Manuel M. The transcription factor Foxg1 regulates the competence of telencephalic cells to adopt subpallial fates in mice. Development (Cambridge, England) 137, 487–497 (2010). 10.1242/dev.039800 20081193 PMC2858907

[8] Hanashima C., Li S. C., Shen L., Lai E. & Fishell G. Foxg1 suppresses early cortical cell fate. Science (New York, N.Y.) 303, 56–59 (2004). 10.1126/science.1090674 14704420

[9] Hanashima C., Shen L., Li S. C. & Lai E. Brain factor-1 controls the proliferation and differentiation of neocortical progenitor cells through independent mechanisms. The Journal of neuroscience : the official journal of the Society for Neuroscience 22, 6526–6536 (2002). 10.1523/jneurosci.22-15-06526.2002 12151532 PMC6758167

[10] Miyoshi G. & Fishell G. Dynamic FoxG1 expression coordinates the integration of multipolar pyramidal neuron precursors into the cortical plate. Neuron 74, 1045–1058 (2012). 10.1016/j.neuron.2012.04.025 22726835 PMC3653132

[11] Cargnin F. FOXG1 Orchestrates Neocortical Organization and Cortico-Cortical Connections. Neuron 100, 1083–1096.e1085 (2018). 10.1016/j.neuron.2018.10.016 30392794 PMC6428593

[12] Kumamoto T. Foxg1 coordinates the switch from nonradially to radially migrating glutamatergic subtypes in the neocortex through spatiotemporal repression. Cell Rep 3, 931–945 (2013). 10.1016/j.celrep.2013.02.023 23523356 PMC3648982

[13] Hou P. S., Miyoshi G. & Hanashima C. Sensory cortex wiring requires preselection of short- and long-range projection neurons through an Egr-Foxg1-COUP-TFI network. Nature communications 10, 3581 (2019). 10.1038/s41467-019-11043-w PMC6687716 31395862

[14] Liu J. FOXG1 sequentially orchestrates subtype specification of postmitotic cortical projection neurons. Sci Adv 8, eabh3568 (2022). 10.1126/sciadv.abh3568 35613274 PMC9132448

[15] Miyoshi G. FoxG1 regulates the formation of cortical GABAergic circuit during an early postnatal critical period resulting in autism spectrum disorder-like phenotypes. Nature communications 12, 3773 (2021). 10.1038/s41467-021-23987-z PMC8213811 34145239

[16] Shen W. Foxg1 Regulates the Postnatal Development of Cortical Interneurons. Cereb Cortex 29, 1547–1560 (2019). 10.1093/cercor/bhy051 29912324 PMC6676970

[17] Elbaz B. & Popko B. Molecular Control of Oligodendrocyte Development. Trends in neurosciences 42, 263–277 (2019). 10.1016/j.tins.2019.01.002 30770136 PMC7397568

[18] Wilpert N. M. Human neuropathology confirms projection neuron and interneuron defects and delayed oligodendrocyte production and maturation in FOXG1 syndrome. Eur J Med Genet 64, 104282 (2021). 10.1016/j.ejmg.2021.104282 34284163

[19] Vegas N. Delineating FOXG1 syndrome: From congenital microcephaly to hyperkinetic encephalopathy. Neurol Genet 4, e281 (2018). 10.1212/nxg.0000000000000281 30533527 PMC6244024

[20] Wong L. C. FOXG1-Related Syndrome: From Clinical to Molecular Genetics and Pathogenic Mechanisms. Int J Mol Sci 20 (2019). 10.3390/ijms20174176 PMC6747066 31454984

[21] Kortum F. The core FOXG1 syndrome phenotype consists of postnatal microcephaly, severe mental retardation, absent language, dyskinesia, and corpus callosum hypogenesis. Journal of medical genetics 48, 396–406 (2011). 10.1136/jmg.2010.087528 21441262 PMC5522617

[22] Cellini E. The hyperkinetic movement disorder of FOXG1-related epileptic-dyskinetic encephalopathy. Dev Med Child Neurol 58, 93–97 (2016). 10.1111/dmcn.12894 26344814

[23] Papandreou A. Delineation of the movement disorders associated with FOXG1 mutations. Neurology 86, 1794–1800 (2016). 10.1212/wnl.0000000000002585 27029630 PMC4862244

[24] Mitter D. FOXG1 syndrome: genotype-phenotype association in 83 patients with FOXG1 variants. Genet Med 20, 98–108 (2018). 10.1038/gim.2017.75 28661489

[25] Hebert J. M. & McConnell S. K. Targeting of cre to the Foxg1 (BF-1) locus mediates loxP recombination in the telencephalon and other developing head structures. Developmental biology 222, 296–306 (2000). 10.1006/dbio.2000.9732 10837119

[26] Eagleson K. L. Disruption of Foxg1 expression by knock-in of cre recombinase: effects on the development of the mouse telencephalon. Neuroscience 148, 385–399 (2007). 10.1016/j.neuroscience.2007.06.012 17640820 PMC2194757

[27] Heinz S. Simple combinations of lineage-determining transcription factors prime cis-regulatory elements required for macrophage and B cell identities. Molecular cell 38, 576–589 (2010). 10.1016/j.molcel.2010.05.004 20513432 PMC2898526

[28] Akol I. Multimodal epigenetic changes and altered NEUROD1 chromatin binding in the mouse hippocampus underlie FOXG1 syndrome. Proceedings of the National Academy of Sciences of the United States of America 120, e2122467120 (2023). 10.1073/pnas.2122467120 36598943 PMC9926245

[29] Huang da W., Sherman B. T. & Lempicki R. A. Systematic and integrative analysis of large gene lists using DAVID bioinformatics resources. Nat Protoc 4, 44–57 (2009). 10.1038/nprot.2008.211 19131956

[30] Kuleshov M. V. Enrichr: a comprehensive gene set enrichment analysis web server 2016 update. Nucleic acids research 44, W90–97 (2016). 10.1093/nar/gkw377 27141961 PMC4987924

[31] Piñero J., Saüch J., Sanz F. & Furlong L. I. The DisGeNET cytoscape app: Exploring and visualizing disease genomics data. Comput Struct Biotechnol J 19, 2960–2967 (2021). 10.1016/j.csbj.2021.05.015 34136095 PMC8163863

[32] Jamwal S. & Kumar P. Insight Into the Emerging Role of Striatal Neurotransmitters in the Pathophysiology of Parkinson’s Disease and Huntington’s Disease: A Review. Curr Neuropharmacol 17, 165–175 (2019). 10.2174/1570159x16666180302115032 29512464 PMC6343208

[33] Menalled L. B. Comprehensive behavioral and molecular characterization of a new knock-in mouse model of Huntington’s disease: zQ175. PLoS One 7, e49838 (2012). 10.1371/journal.pone.0049838 23284626 PMC3527464

[34] Ferrari Bardile C. Intrinsic mutant HTT-mediated defects in oligodendroglia cause myelination deficits and behavioral abnormalities in Huntington disease. Proceedings of the National Academy of Sciences of the United States of America 116, 9622–9627 (2019). 10.1073/pnas.1818042116 31015293 PMC6511031

[35] Erickson K. R. Behavioral and brain anatomical analysis of Foxg1 heterozygous mice. PLoS One 17, e0266861 (2022). 10.1371/journal.pone.0266861 36223387 PMC9555627

[36] Younger S. Behavioral Phenotypes of Foxg1 Heterozygous Mice. Front Pharmacol 13, 927296 (2022). 10.3389/fphar.2022.927296 35754477 PMC9214218

[37] Bey A. L. & Jiang Y. H. Overview of mouse models of autism spectrum disorders. Curr Protoc Pharmacol 66, 5.66.61–65.66.26 (2014). 10.1002/0471141755.ph0566s66 PMC4186887 25181011

[38] Siegenthaler J. A., Tremper-Wells B. A. & Miller M. W. Foxg1 haploinsufficiency reduces the population of cortical intermediate progenitor cells: effect of increased p21 expression. Cereb Cortex 18, 1865–1875 (2008). 10.1093/cercor/bhm209 18065723 PMC2790389

[39] Budimirovic D. B. & Kaufmann W. E. What can we learn about autism from studying fragile X syndrome? Dev Neurosci 33, 379–394 (2011). 10.1159/000330213 21893949 PMC3254037

[40] Cochet-Bissuel M., Lory P. & Monteil A. The sodium leak channel, NALCN, in health and disease. Front Cell Neurosci 8, 132 (2014). 10.3389/fncel.2014.00132 24904279 PMC4033012

[41] Montani C., Gritti L., Beretta S., Verpelli C. & Sala C. The Synaptic and Neuronal Functions of the X-Linked Intellectual Disability Protein Interleukin-1 Receptor Accessory Protein Like 1 (IL1RAPL1). Dev Neurobiol 79, 85–95 (2019). 10.1002/dneu.22657 30548231

[42] Ropers H. H. X-linked mental retardation: many genes for a complex disorder. Curr Opin Genet Dev 16, 260–269 (2006). 10.1016/j.gde.2006.04.017 16647850

[43] Saini A. G., Sankhyan N. & Vyas S. PLEKHG2-associated neurological disorder. BMJ Case Rep 14 (2021). 10.1136/bcr-2021-244206 PMC8323387 34326120

[44] Edvardson S. Hereditary sensory autonomic neuropathy caused by a mutation in dystonin. Ann Neurol 71, 569–572 (2012). 10.1002/ana.23524 22522446

[45] Cossée M. ARX polyalanine expansions are highly implicated in familial cases of mental retardation with infantile epilepsy and/or hand dystonia. American journal of medical genetics. Part A 155a, 98–105 (2011). 10.1002/ajmg.a.33785 21204215

[46] Goizet C. Progressive bulbospinal amyotrophy in triple A syndrome with AAAS gene mutation. Neurology 58, 962–965 (2002). 10.1212/wnl.58.6.962 11914417

[47] Mencacci N. E. A missense mutation in KCTD17 causes autosomal dominant myoclonus-dystonia. American journal of human genetics 96, 938–947 (2015). 10.1016/j.ajhg.2015.04.008 25983243 PMC4457957

[48] Turnbull J. Early-onset Lafora body disease. Brain 135, 2684–2698 (2012). 10.1093/brain/aws205 22961547 PMC3437029

[49] Marcello E., Di Luca M. & Gardoni F. Synapse-to-nucleus communication: from developmental disorders to Alzheimer’s disease. Curr Opin Neurobiol 48, 160–166 (2018). 10.1016/j.conb.2017.12.017 29316492

[50] Kumar A. & Sharma S. in StatPearls (StatPearls Publishing Copyright © 2023, StatPearls Publishing LLC., 2023).

[51] Blumenfeld H. Arousal and Consciousness in Focal Seizures. Epilepsy Curr 21, 353–359 (2021). 10.1177/15357597211029507 34924835 PMC8655266

[52] Mohamed J., Scott B. W., David O. & McIntyre Burnham W. Development of propagated discharge and behavioral arrest in hippocampal and amygdala-kindled animals. Epilepsy Res 148, 78–89 (2018). 10.1016/j.eplepsyres.2018.10.010 30391634

[53] Fisher R. S. The New Classification of Seizures by the International League Against Epilepsy 2017. Curr Neurol Neurosci Rep 17, 48 (2017). 10.1007/s11910-017-0758-6 28425015

[54] Seltzer L. E. Epilepsy and outcome in FOXG1-related disorders. Epilepsia 55, 1292–1300 (2014). 10.1111/epi.12648 24836831 PMC4265461

[55] Brancaccio M., Pivetta C., Granzotto M., Filippis C. & Mallamaci A. Emx2 and Foxg1 inhibit gliogenesis and promote neuronogenesis. Stem Cells 28, 1206–1218 (2010). 10.1002/stem.443 20506244

[56] Dong F. Conditional Deletion of Foxg1 Alleviates Demyelination and Facilitates Remyelination via the Wnt Signaling Pathway in Cuprizone-Induced Demyelinated Mice. Neuroscience bulletin 37, 15–30 (2021). 10.1007/s12264-020-00583-7 33015737 PMC7811968

[57] Fields R. D. White matter in learning, cognition and psychiatric disorders. Trends in neurosciences 31, 361–370 (2008). 10.1016/j.tins.2008.04.001 18538868 PMC2486416

[58] Dou C. L., Li S. & Lai E. Dual role of brain factor-1 in regulating growth and patterning of the cerebral hemispheres. Cereb Cortex 9, 543–550 (1999). 10.1093/cercor/9.6.543 10498272

[59] Hardcastle Z. & Papalopulu N. Distinct effects of XBF-1 in regulating the cell cycle inhibitor p27(XIC1) and imparting a neural fate. Development (Cambridge, England) 127, 1303–1314 (2000). 10.1242/dev.127.6.1303 10683182

[60] Lu W. NFIA haploinsufficiency is associated with a CNS malformation syndrome and urinary tract defects. PLoS genetics 3, e80 (2007). 10.1371/journal.pgen.0030080 17530927 PMC1877820

[61] Mikhail F. M. Clinically relevant single gene or intragenic deletions encompassing critical neurodevelopmental genes in patients with developmental delay, mental retardation, and/or autism spectrum disorders. American journal of medical genetics. Part A 155a, 2386–2396 (2011). 10.1002/ajmg.a.34177 22031302

[62] Schanze I. NFIB Haploinsufficiency Is Associated with Intellectual Disability and Macrocephaly. American journal of human genetics 103, 752–768 (2018). 10.1016/j.ajhg.2018.10.006 30388402 PMC6218805

[63] Dobin A. STAR: ultrafast universal RNA-seq aligner. Bioinformatics 29, 15–21 (2013). 10.1093/bioinformatics/bts635 23104886 PMC3530905

[64] Li B. & Dewey C. N. RSEM: accurate transcript quantification from RNA-Seq data with or without a reference genome. BMC Bioinformatics 12, 323 (2011). 10.1186/1471-2105-12-323 21816040 PMC3163565

[65] Leng N. EBSeq: an empirical Bayes hierarchical model for inference in RNA-seq experiments. Bioinformatics 29, 1035–1043 (2013). 10.1093/bioinformatics/btt087 23428641 PMC3624807

